# Environmental conditions differentially affect neurobehavioral outcomes in a mouse model of sepsis-associated encephalopathy

**DOI:** 10.18632/oncotarget.19595

**Published:** 2017-07-26

**Authors:** Mu-Huo Ji, Hui Tang, Dan Luo, Li-Li Qiu, Min Jia, Hong-Mei Yuan, Shan-Wu Feng, Jian-Jun Yang

**Affiliations:** ^1^ Department of Anesthesiology, Zhongda Hospital, Medical School, Southeast University, Nanjing, China; ^2^ Department of Anesthesiology, Jinling Hospital, School of Medicine, Nanjing University, Nanjing, China; ^3^ Department of Anesthesiology, Obstetrics and Gynecology Hospital Affiliated to Nanjing Medical University, Nanjing, China

**Keywords:** sepsis, environmental conditions, neuroplasticity, cognitive function

## Abstract

Brain dysfunction remains a common complication after sepsis development and is an independent risk factor for a poorer prognosis and an increased mortality. Here we tested the hypothesis that the behavioral outcomes after lipopolysaccharides (LPS) administration are exacerbated by an impoverished environment (IE) and alleviated by an enriched environment (EE), respectively. Mice were randomly allocated in a standard environment (SE), an EE, or an IE for 4 weeks after LPS or normal saline (NS) administration. Neurobehavioral alternations were assessed by the open field, novel objective recognition, and fear conditioning tests. The expressions of proinflammatory cytokines (tumor necrosis factor (TNF-α), interleukin-1β (IL-1β), IL-6, IL-10), ionized calcium-binding adaptor molecule-1 (IBA1)-positive cells as well as glial fibrillary acidic protein (GFAP)-positive cells, brain derived neurotrophic factor (BDNF), 5-bromo-2-deoxyuridine-labeled cells in the dentate gyrus of the hippocampus, and the number of dendritic spines in the hippocampal CA1 were determined. Our results showed that the some of the neurocognitive abnormalities induced by LPS administration can be aggravated by stressful conditions such as IE but alleviated by EE. These neurocognitive alternations were accompanied by significant changes in biomarkers of immune response and hippocampal synaptic plasticity. In summary, our study confirmed the negative impact of IE and the positive effects of EE on the cognitive function after LPS administration, with potential implications to the basis of sepsis-related cognitive impairments in the critically ill patients.

## INTRODUCTION

Sepsis is a major clinical challenge associated with multi-organ dysfunction, including sepsis associated-encephalopathy (SAE) [1–3]. Studies from rodent animals suggest that sepsis impairs learning and memory functions in animal models of SAE induced by peripheral administration of lipopolysaccharides (LPS) or cecal ligation and puncture (CLP) [4–6]. SAE is associated with an increased risk for the development of mental, cognitive, and physical impairments that may persist for months or years after hospital discharge [1–3]. With recent advances in critical care medicine, the chance of survival after critical illness has increased tremendously. As a result, patients often require long-term medical interventions to support functional recovery after sepsis development. The negative impact of the long-term neurobehavioral consequences includes reduced quality of life, poor prognosis, and increased mortality [1–3].

Accumulating evidence has shown that environmental conditions affect cognitive performance in many neuropsychiatric disorders in animal and human studies [7–12]. An enriched environment (EE) is defined as a manipulation that increases physical and social stimuli [8], while an impoverished environment (IE) offers standard cages with reduced sensorial and cognitive stimulation [11]. It is well recognized that EE can improve spatial learning, enhance long-term memory, and prevent cognitive deficits induced by stress [8, 9], whereas IE alter behavioral and neuroendocrine response with negative outcomes when compared with group housing [11, 12]. These findings strongly suggested that environmental conditions may also modulate the outcomes of long-term cognitive consequences associated with sepsis.

Patients living in the intensive care unit are often associated with an IE, which may have negative effects on cognitive functioning [13–15]. However, few studies have proven that environmental conditions can affect sepsis-induced cognitive impairment. In the present study, we hypothesized that sepsis-induced long-term cognitive impairments can been improved by EE and aggravated by IE, respectively.

## RESULTS

### Effects of LPS and environmental conditions on body weight gain and survival rate

**Figure 1:**
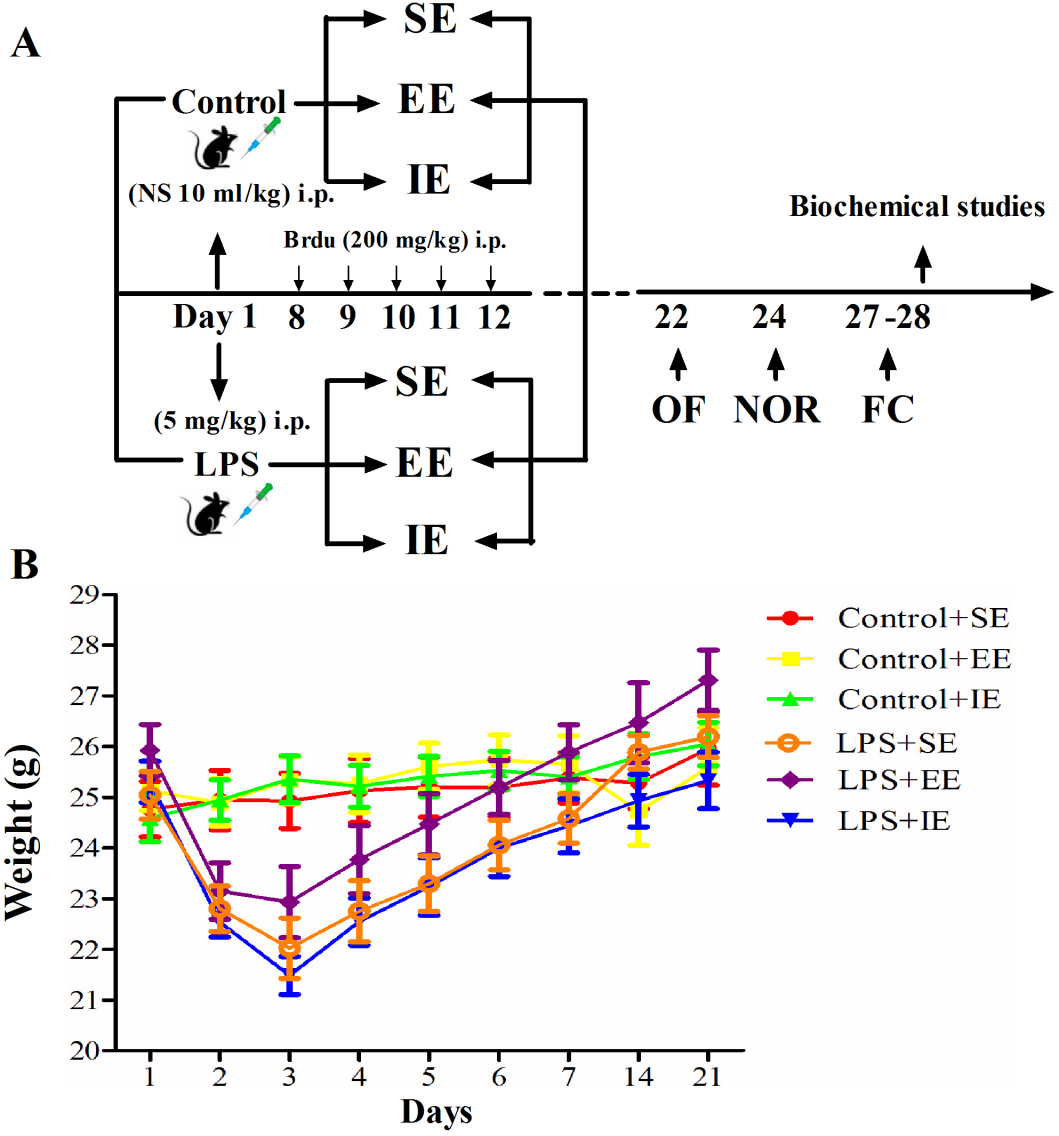
Timeline of the experimental procedure in mice **(A).** Effects of LPS and environmental conditions on body weight gain **(B).** SE, standard environment; EE, enriched environment; IE, impoverished environment; OF, open field; NOR, novel object recognition; FC, fear conditioning. NS, normal saline; LPS, lipopolysaccharide.

As shown in Figure 1B, LPS administration resulted in body weight loss for about 2 days when compared with baseline values. However, environmental conditions did not affect body weight gain among the NS exposed groups or LPS exposed groups (two-way repeated measures ANOVA; *P* = 0.7326).

There was no death for the NS exposed mice. In addition, LPS or environmental conditions did not affect the 7-day survival rate for the LPS-exposed mice (93.7% for LPS + SE group, 87.5% for LPS + EE group, and 86.667% for LPS + IE group, *P* = 0.4568).

### Effects of LPS and environmental conditions on anxiety-like behavior

**Figure 2:**
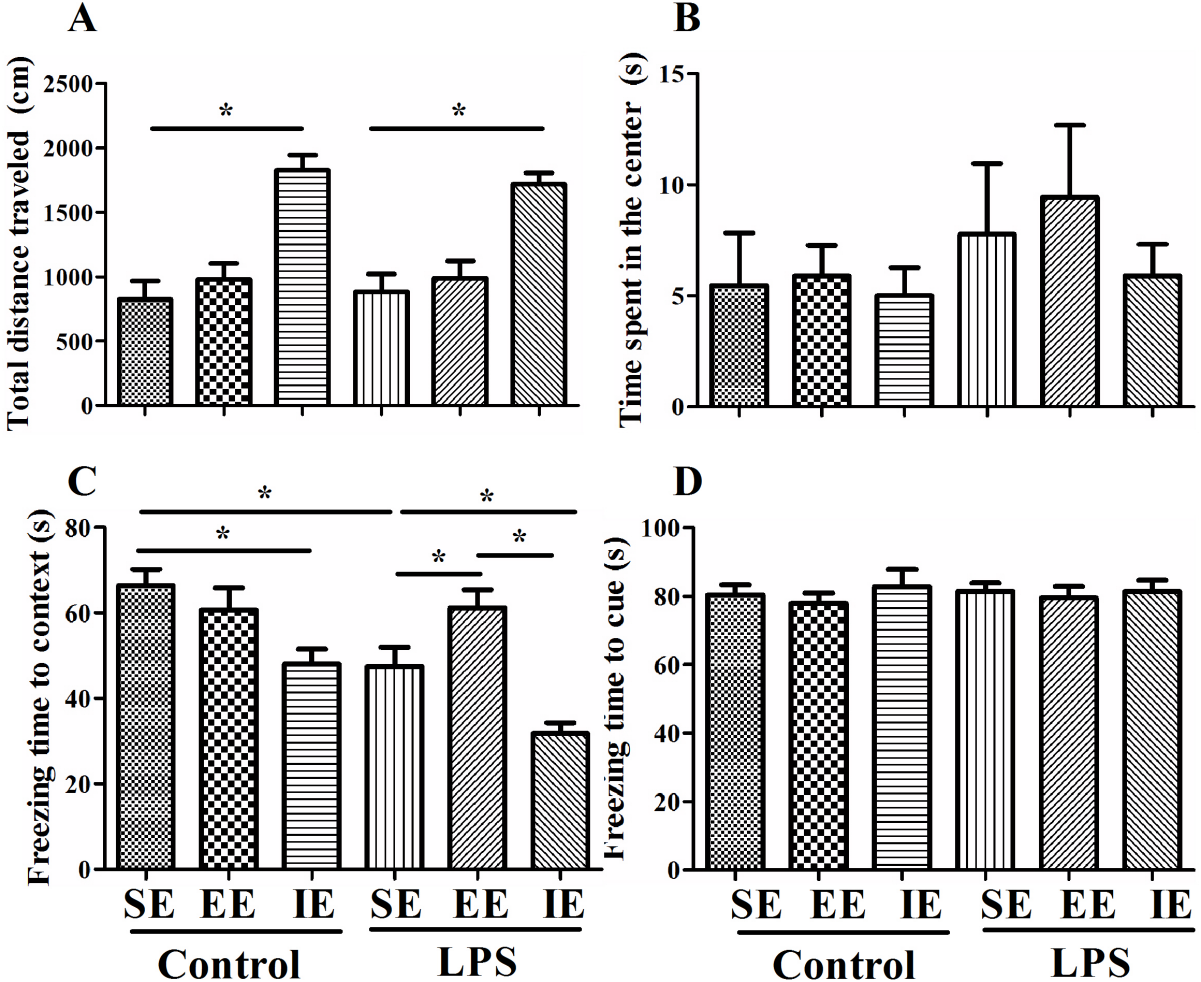
Effects of LPS and environmental conditions on anxiety-like behavior and fear conditioning response Open field test was performed to examine the anxiety-like behavior and locomotion **(A** and **B)**. LPS-exposed mice displayed an environment-dependent impairment in cognitive function **(C** and **D)**. SE, standard environment; EE, enriched environment; IE, impoverished environment. Each value represents the mean ± SEM (n =11-14 per group), *P < 0.05.

As shown in Figure 2A and 2B, LPS exposed mice and NS exposed mice in the IE condition traveled significantly longer distance in the open arena when compared with their control counterparts housed in the SE condition (F_(5, 67)_ = 13.333, *P* < 0.001), suggesting IE induced anxiety-like behavior. However, EE did not reverse the increased travel distance in the control + EE and LPS + EE groups. There was no significant difference in the time spent in the center among the six groups (F_(5, 67)_ = 0.525, *P* = 0.757).

### Effects of LPS and environmental conditions on social interaction

**Figure 3:**
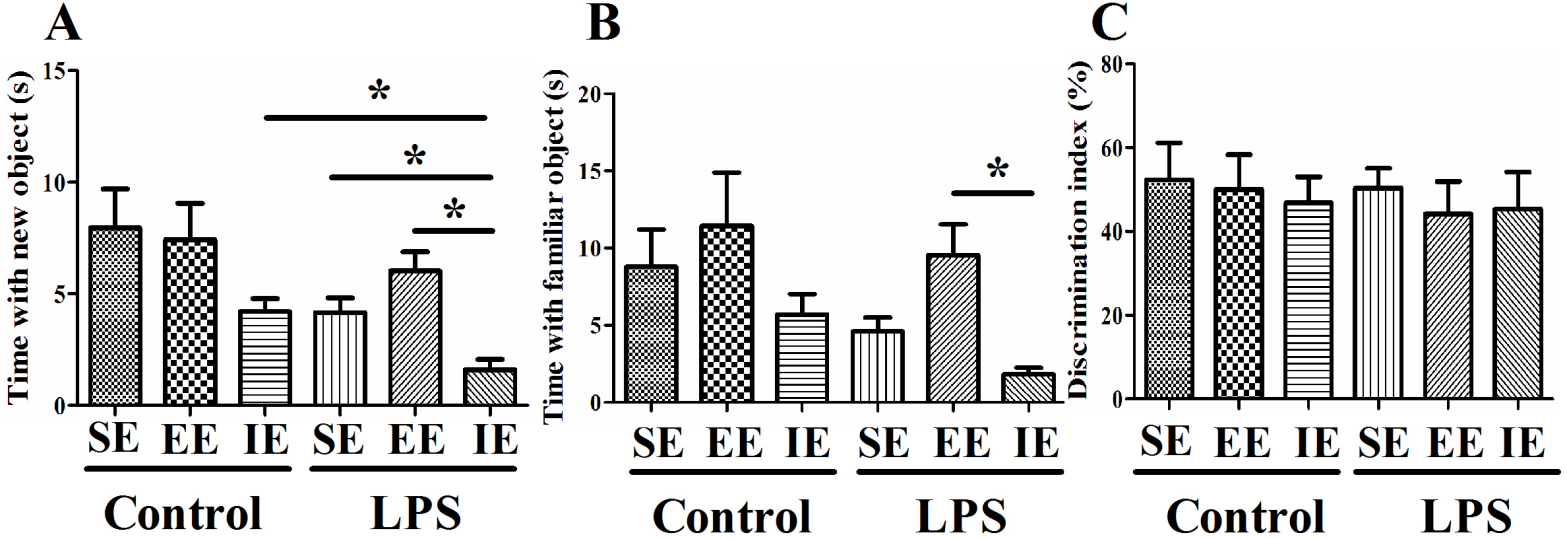
Effects of LPS and environmental conditions on social interaction LPS exposed mice housed in IE condition explored both the familiar and the novel object significantly less than the control + IE group **(A** and **B)**. There was no significant difference in discrimination index among groups **(C)**. SE, standard environment; EE, enriched environment; IE, impoverished environment. Each value represents the mean ± SEM (n =10-12 per group), *P < 0.05.

The social interaction test is used to assess working memory as well as generalized exploration behavior. Although there was no difference between the NS and LPS exposed mice in their exploration of the novel object compared to the familiar object, we found that LPS exposed mice housed in the IE condition explored both the familiar and the novel object significantly less than the control + IE group (novel, *P* = 0.002; familiar, *P* = 0.022; Figure 3A and 3B). In addition, the LPS exposed mice housed in the IE also explored the novel object significantly less than the LPS + SE group or LPS + EE group. However, EE failed to reverse these behavioral abnormities. In addition, there was no difference in preference index among groups (F_(5, 60)_ = 0.176, *P* = 0.971; Figure 3C).

### Effects of LPS and environmental conditions on fear conditioning response

The NS exposed mice housed in the IE condition exhibited decreased freezing time in the contextual fear conditioning compared with the control + SE group. LPS exposed mice also displayed significant decline in the freezing time in the contextual fear conditioning compared with the control + SE group. Interestingly, LPS exposed mice housed in the IE condition further decreased the freezing time in the contextual fear conditioning compared with the LPS + SE group, but mice housed in EE reversed LPS-induced cognitive impairment (F_(5, 68)_ = 12.135, *P* < 0.001, Figure 2C). These results suggested that LPS exposed mice displayed an environment-dependent impairment in fear conditioning response. However, there was no difference in the freezing time in the hippocampal-independent cued test among groups (F_(5, 68)_ = 0.235, *P* = 0.946, Figure 2D).

### Effects of LPS and environmental conditions on brain inflammatory responses

**Figure 4:**
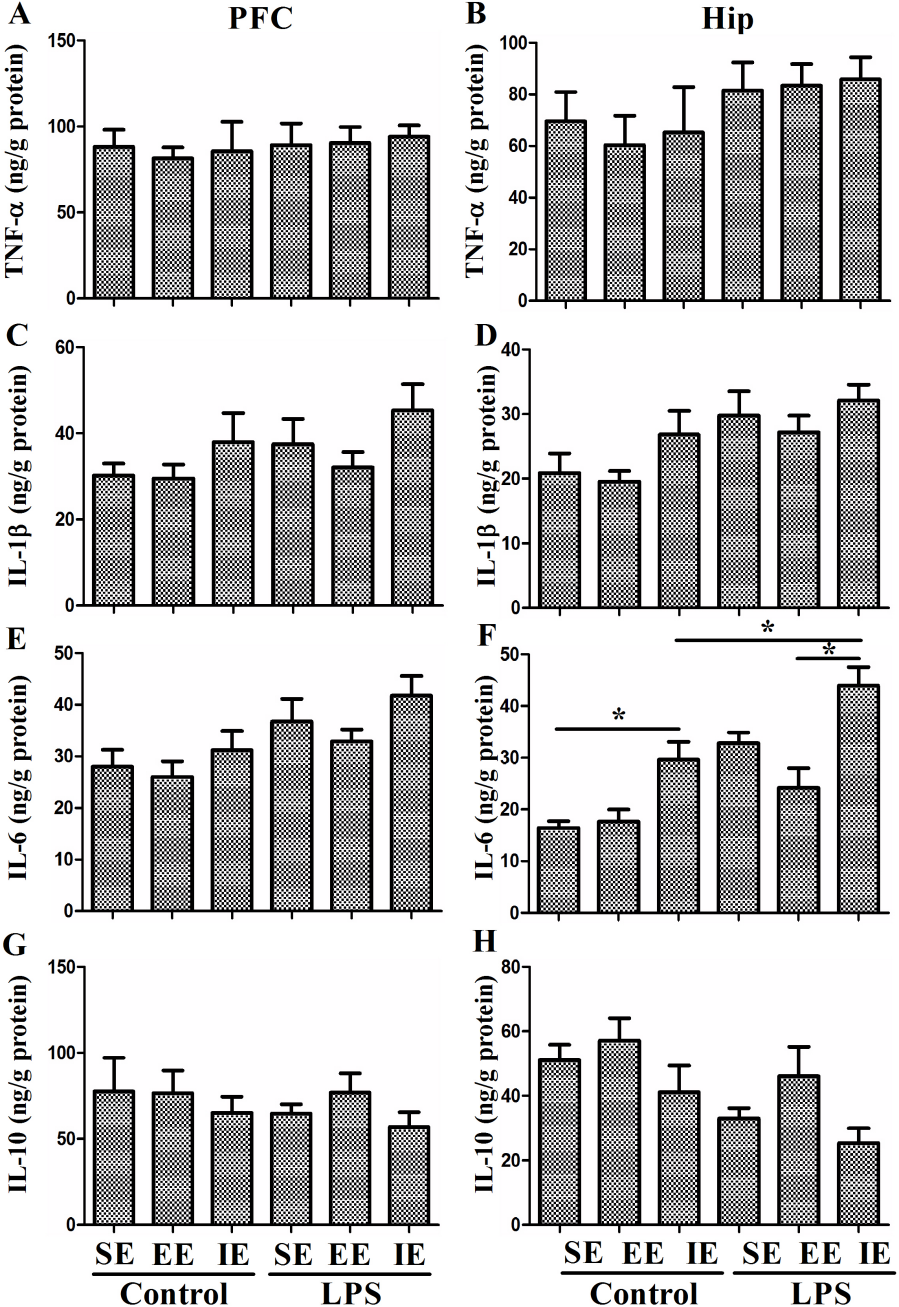
Effects of LPS and environmental conditions on brain inflammatory responses No difference was observed in TNF-α **(A)**, IL-1β **(C)**, IL-6 **(E)**, and IL-10 **(G)** expressions in the prefrontal cortex among groups. In the hippocampus, the NS exposed mice housed in the IE condition had significantly higher level of IL-6 compared with the control + SE group. LPS exposed mice also displayed significantly higher level of IL-6 compared with the control + SE group. Notably, LPS exposed mice housed in the IE condition further increased IL-6 expression compared with LPS+SE group. There was no significant difference in hippocampal TNF-α, IL-1β, and IL-10 levels among the six groups **(B, D, F, H)**. SE, standard environment; EE, enriched environment; IE, impoverished environment. Each value represents the mean ± SEM (n =5 per group), *P < 0.05.

To evaluate the effects of LPS and environmental conditions on brain inflammatory responses, we measured TNF-α, IL-1β, IL-6, and IL-10 expressions in the prefrontal cortex and hippocampus at 28 day after NS or LPS administration. As shown in Figure 4, neither LPS administration nor environmental conditions affected TNF-α, IL-1β, IL-6, and IL-10 expressions in the prefrontal cortex (TNF-α: F_(5, 24)_ = 0.154, *P* = 0.977; IL-1β: F_(5, 24)_ = 1.468, *P* = 0.237; IL-6: F_(5, 24)_ = 2.761, *P* = 0.042; IL-10: F_(5, 24)_ = 0.519, *P* = 0.760). In the hippocampus, the NS exposed mice housed in the IE condition had significantly higher level of IL-6 compared with the control + SE group. LPS exposed mice also displayed significantly higher level of IL-6 compared with the control + SE group. Notably, LPS exposed mice housed in the IE condition further increased IL-6 expression compared with the LPS+SE group (F_(5, 24)_ = 12.571, *P* < 0.001). However, there was no significant difference in hippocampal TNF-α, IL-1β, and IL-10 levels among the six groups (TNF-α: F_(5, 24)_ = 0.818, *P*=0.549; IL-1β: F_(5, 24)_ = 2.794, *P* = 0.04; IL-10: F_(5, 24)_ = 3.243, *P* = 0.022).

**Figure 5:**
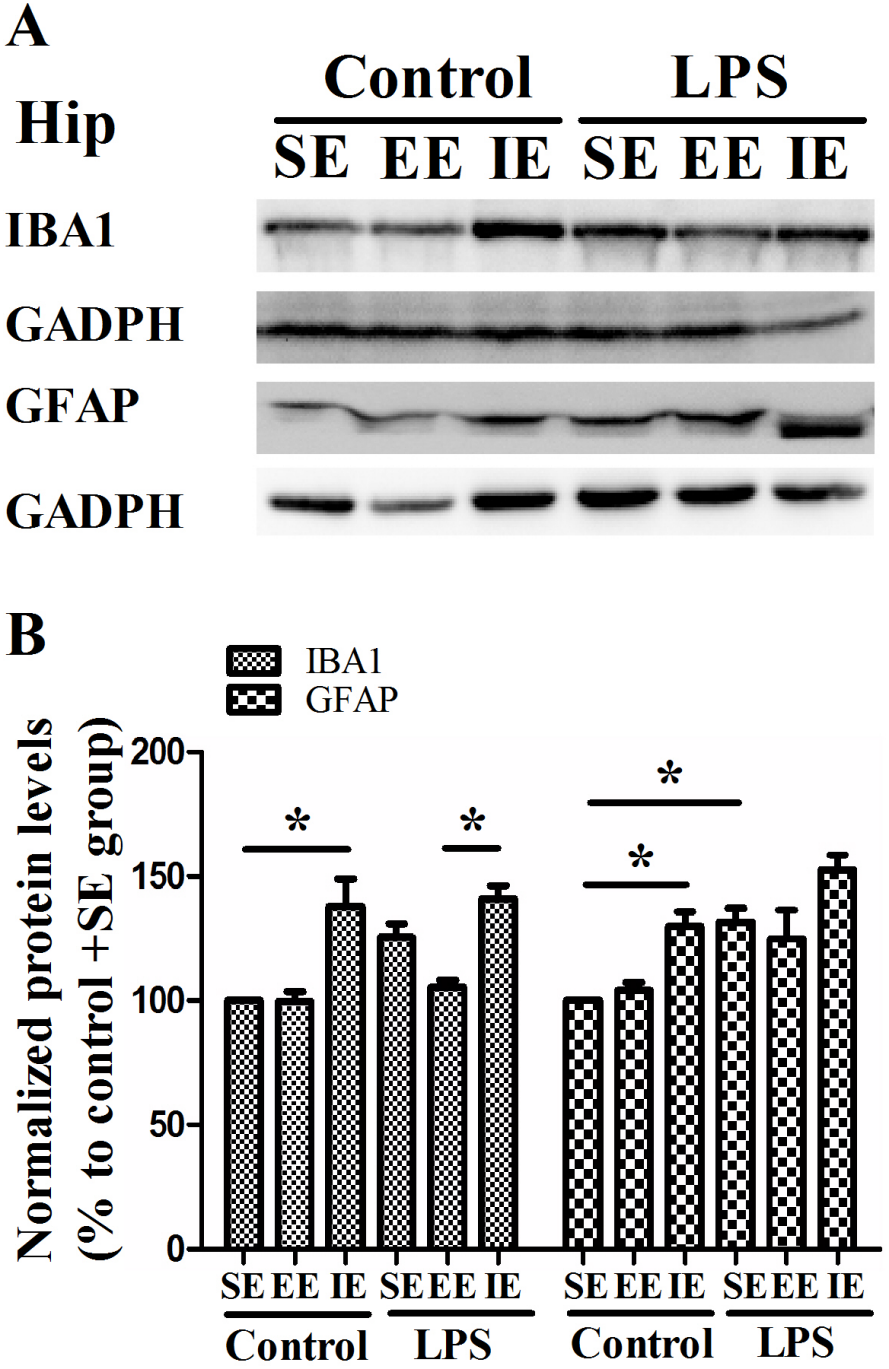
Effects of LPS and environmental conditions on hippocampal IBA1 and GFAP expressions Representative images of IBA1 and GFAP in the hippocampus **(A)**. Quantification of IBA1 and GFAP expressions in the hippocampus **(B)**. SE, standard environment; EE, enriched environment; IE, impoverished environment. Each value represents the mean ± SEM (n =4 per group), *P < 0.05.

As shown in Figure 5, the NS exposed mice housed in the IE condition showed significantly increased hippocampal IBA1 expression compared with the control + SE group. Unexpectedly, LPS exposure did not result in higher expression of IBA1 compared with the control + SE group (F_(5, 18)_ = 10.142, *P* < 0.001). In addition, the LPS exposed mice housed in the IE condition did not further increase the level of IBA1 compared with the LPS + SE group. As for the GFAP, the NS exposed mice housed in the IE condition showed significantly increased GFAP expression compared with the control + SE group. However, LPS exposed mice housed in the SE also had significantly higher level of GFAP compared with the control + SE group (F_(5, 18)_ = 9.211, *P* < 0.001).

### Effects of LPS and environmental conditions on hippocampal neuronal proliferation

**Figure 6:**
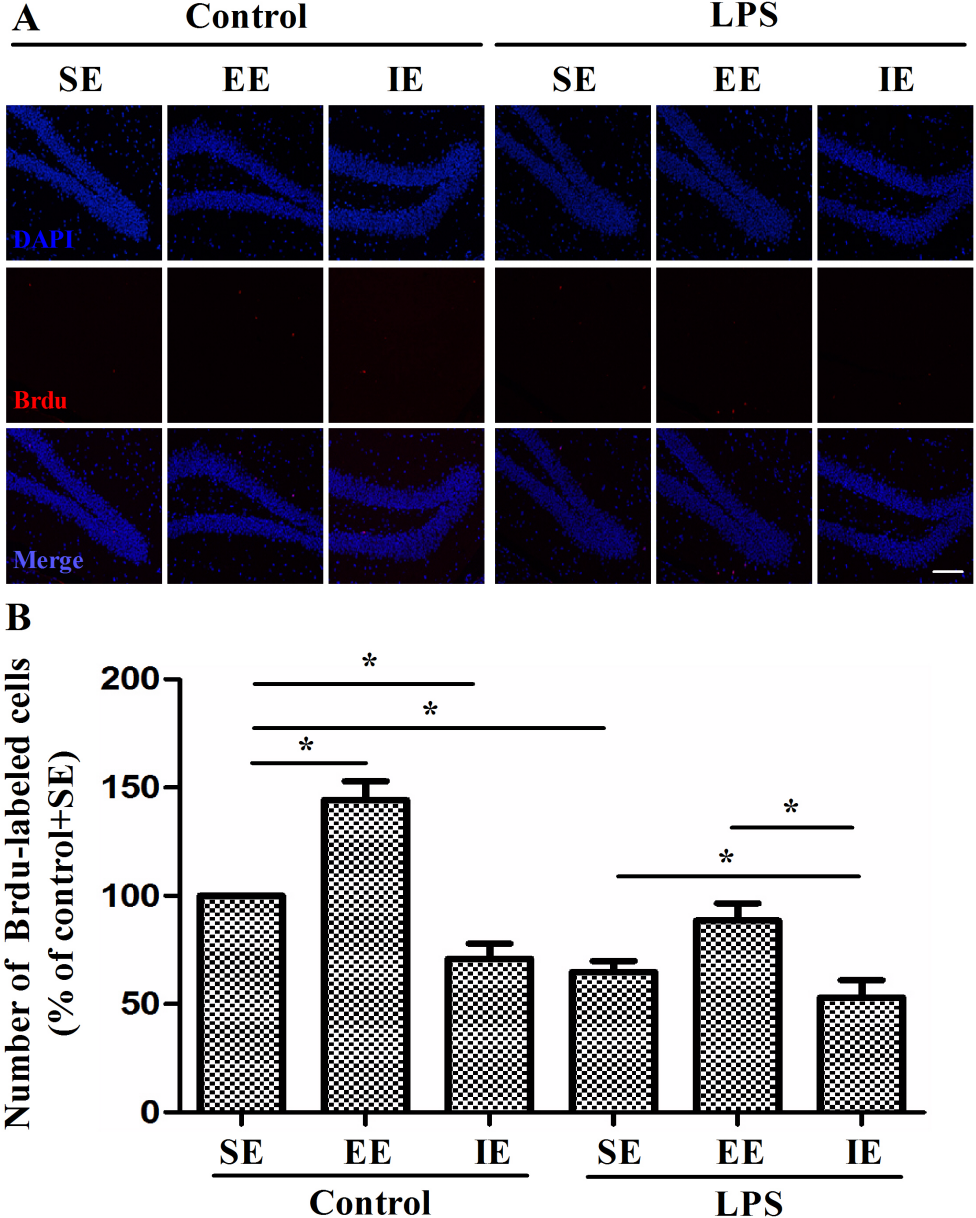
Effects of LPS and environmental conditions on hippocampal neuronal proliferation Representative images of BrdU cells at the DG region by immunofluorescence **(A)**. Quantification of BrdU at the DG region of hippocampus **(B)**. SE, standard environment; EE, enriched environment; IE, impoverished environment. Each value represents the mean ± SEM (n = 6 per group), *P < 0.05; scale bar = 50 μm.

Newly generated BrdU-labeled cells were observed in the DG by immunofluorescence labeling. The NS exposed mice housed in the IE condition showed decreased number of BrdU-labeled cells in the DG compared with the control + SE group. Likewise, LPS exposed mice showed significantly decreased BrdU-labeled cells in the DG compared with the control + SE group. In addition, the LPS exposed mice housed in the IE further decreased the number of BrdU-labeled cells in the DG compared with the LPS+SE group (F_(5, 30)_ = 22.983, *P* < 0.001, Figure 6). Although EE was able to reverse IE-induced neuronal proliferation inhibition for the NS exposed mice, it did not completely reverse the decrease in BrdU-positive cells for LPS exposed mice.

### Effects of LPS and environmental conditions on dendritic spine in the hippocampal CA1

**Figure 7:**
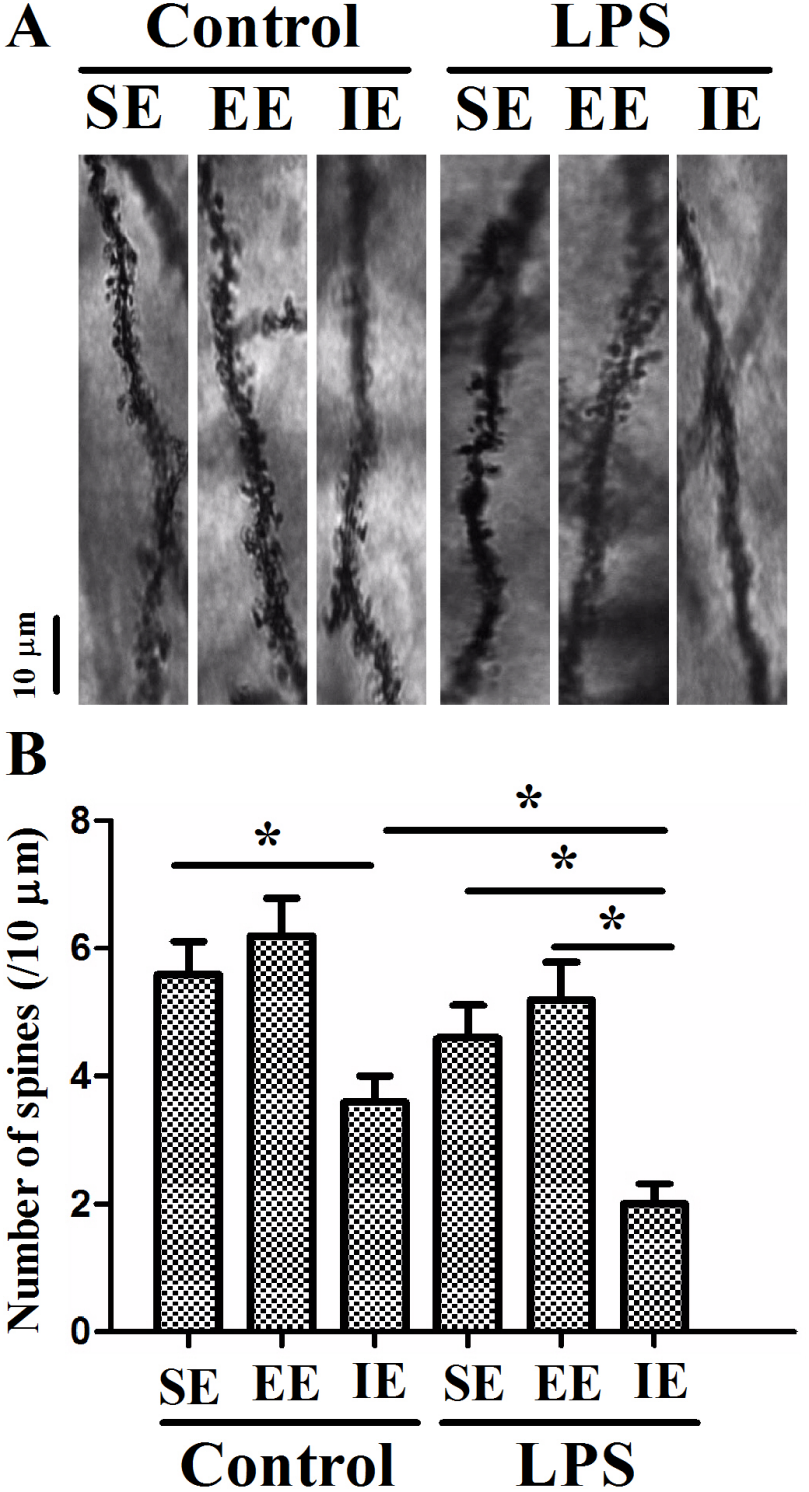
Effects of LPS and environmental conditions on dendritic spine in the hippocampal CA1 Representative images of dendritic segments of pyramidal neurons in the hippocampus **(A)**. Histograms represented the number of dendritic spines/10 μm length of primary dendrites **(B)**. SE, standard environment; EE, enriched environment; IE, impoverished environment. Each value represents the mean ± SEM (n =5 per group), *P < 0.05.

As revealed in Figure 7, the number of dendritic spines in the hippocampal CA1 region decreased significantly in the LPS + SE group compared with the control + SE group, suggesting that LPS-induced cognitive impairment was associated with decreased dendritic spine number. In addition, LPS exposed mice housed in the IE condition further aggravated the decrease in dendritic spine number compared with the LPS + SE group (F_(5, 24)_ = 9.578, *P* < 0.001). Again, EE did not significantly attenuate the decreased dendritic spine number in the hippocampal CA1 region.

### Effects of LPS and environmental conditions on hippocampal neurotrophic factors

**Figure 8:**
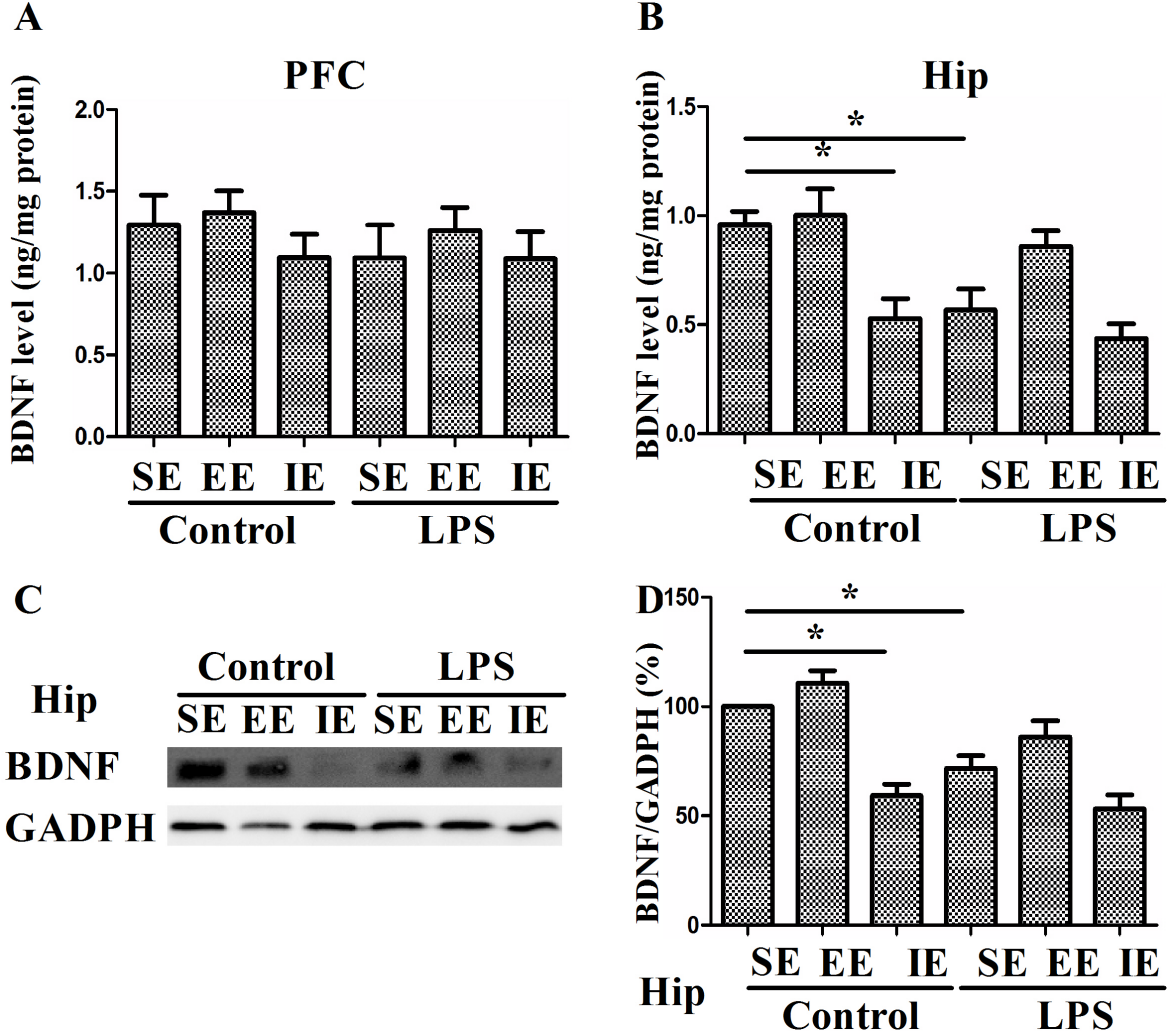
Effects of LPS and environmental conditions on BDNF expressions BDNF levels were assessed by ELISA in the prefrontal cortex and hippocampus **(A** and **B)**. Representative image of BDNF was assessed by western blotting **(C)**. Histograms represent the levels of BDNF **(D)**. SE, standard environment; EE, enriched environment; IE, impoverished environment. Each value represents the mean ± SEM (n =4-5 per group), *P < 0.05.

As shown in Figure 8, the level of BDNF decreased significantly in the control + IE group compared with those in the control + SE group, suggesting IE impaired cognitive impairment by decreasing BDNF level in the hippocampus (F(_5, 24)_ = 8.833, *P* < 0.001, Figure 8A) but not in the prefrontal cortex (F_(5, 24)_ = 0.575, *P* = 0.719, Figure 8B). Similarly, LPS exposed mice in the SE had reduced BDNF level as compared with the control + SE group. However, either EE or IE condition did not increase or further decrease BDNF levels, respectively. The similar results were confirmed by the western blot analysis (F_(5, 18)_ = 16.394, *P* < 0.001, Figure 8C).

## DISCUSSION

In the present study, we showed that the neurobehavioral outcomes after LPS administration are affected by subsequent environmental conditions, with EE alleviating and IE exacerbating the cognitive impairment, respectively. The mechanisms underlying these behavioral changes may be related to the cellular effects on immune responses and hippocampal synaptic plasticity. Thus, interventions that improving environmental condition may produce better long-term neurobehavioral outcomes.

Although the brain may be affected during early sepsis, it has only recently been shown that the presence of brain dysfunction is an independent factor for the poor prognosis in patients with sepsis [1–3]. Environmental factors have significant impacts on the behavioral outcomes in many psychiatric diseases. For example, high levels of social or physical activity reduce the risk of Alzheimer’s disease [16]. In major depression, social support has the beneficial effects on the outcome of a depressive episode and prevention of relapse [17]. In preclinical study, it has been shown that animals housed in the EE condition exhibit increases in brain size, the number of neurons in the DG, the number and area of synapses, and the production of neurotrophic factors compared with those housed in the IE condition [18]. In comparison, sedentary and lonely people present worse cognitive functions and show a faster cognitive decline than physically and socially active people [19]. Notably, the effects of environmental conditions on cognitive function are reversible, with improvement in cognitive performance after old animals being transferred from an IE into an EE condition [7].

In our study, the mice housed in the IE condition exhibited anxiety-like behavior, but among these mice, only the LPS-exposed mice showed reduced time in their exploration of the novel object. It has been previously demonstrated that impairment in communication and social interaction are major symptoms of autism spectrum disorders, a group of common neuropsychiatric disorders in which environmental factors are thought to play an etiological role [20, 21]. In addition, LPS-exposed mice displayed an environment-dependent impairment in fear conditioning response. Consequently, the behavioral abnormalities induced by LPS in combination with a stressful environment such as IE may be even greater, suggesting these two factors have synergistic effects on neurobehavioral outcomes. Intriguingly, our study showed that LPS exposure did not cause significant decline in social interaction, which is partly supported by previous finding that LPS administration does not impair cognition in all hippocampus-dependent tasks [22]. In addition, we found that EE affected only some of the behavioral functions, which might attributed to the relatively short period of EE exposure. Our results are in line with one prior study demonstrating that EE requires long duration to exert beneficial effects on cognitive aging [23, 24]. Although EE does not reverse all the neurocognitve impairment, it can be considered as a social support, which could prevent cognitive impairment by LPS administration. On the other hand, our study also indicated that IE can be served as a social stress that further aggregates cognitive impairment induced by LPS injection. However, the mechanism underlying these effects needs to be elucidated.

There is a high density of cytokine receptors in the hippocampus, suggesting the hippocampus may be vulnerable during neuroinflammation [25]. Although physiological level of cytokines is necessary for synaptic plasticity and neurotransmission, it is well established that overproduction of these inflammatory mediators may negatively affect neuronal processes pertinent to cognition [26, 27]. Concurrent with cognitive impairment, our study showed that LPS-exposed mice had significantly higher level of hippocampal inflammatory cytokine (IL-6). Importantly, hippocampal IL-6 expression was further increased in the mice housed in the IE condition. Although IL-6 was not totally reversed by the EE, our study suggested the importance of environmental conditions on the immune system function specifically within the hippocampus, which might have profound effects on neural function. Indeed, it has been suggested that social interaction reduces post-stroke depression and improves functional recovery by down-regulating hippocampal IL-6 level in an animal model of stroke [28]. Previous studies have shown that elevated levels of proinflammatory cytokines in the hippocampus inhibit neurotrophic factors and impair hippocampal synaptic plasticity [29, 30], providing a potential mechanism whereby hippocampal neuroinflammation can induce cognitive impairment. Consistently, our data suggested that LPS-exposed mice housed in the IE condition had the highest level of hippocampal inflammatory cytokine (IL-6) and lowest hippocampal BDNF level. This is further supported by the increased hippocampal expressions of microglia marker IBA1 and astrocyte marker GFAP in mice housed in the IE. Therefore, we speculated that the loss of neurotrophic support along with the dysregulated neuron-glia crosstalk could have significant negative impact on cognitive function and render the brain vulnerable to inflammatory damage during sepsis development.

Animal studies have demonstrated that the effects of environmental conditions on learning and memory were associated with structural and functional changes in the hippocampus, such as synaptic and spine density, neurogenesis, and long-term potentiation [31–33]. Accumulating evidence has shown that neurotrophic factors play an important role in learning, memory, and hippocampal plasticity [29, 30]. In one previous study, the LPS-induced reduction in hippocampal BDNF could have deleterious effects on cognitive function after sepsis development [30]. Although the IE did not further decreased BDNF level in LPS-exposed mice, we did find that the IE paradigm decreased basal levels of BDNF in the hippocampus. By contrast, EE has been shown to exert positive effects on cognitive and emotional behaviors including enhanced learning and memory [34] and improved ability to cope with fear, anxiety, and stress [35]. Since BDNF is known to specifically impact hippocampal-dependent tasks [32], the different environmental condition-induced modulation of BDNF could help to influence cognitive function during sepsis development. Specifically, the EE has been shown to increase BDNF expression, markers of synaptic plasticity that is critically involved in the learning [8]. In the present study, we did not find any significant difference in the levels of synaptic markers such as BDNF and dendritic spines in the hippocampus between the LPS+SE and the LPS+EE groups, but we found that LPS-exposed mice, after being housed in the IE for 4 weeks, showed a significantly lower levels of BDNF and dendritic spines. This loss of neurotrophic support could help to explain why the IE even has greater negative impact on cognitive function than only one LPS administration.

In addition, there is accumulating evidence suggesting that adult neurogenesis is closely linked to hippocampal-dependent learning and memory [32]. It has been shown that factors that increase adult neurogenesis, such as EE and exercise, improve memory function, whereas a reduction of neurogenesis induced by stress leads to cognitive impairment [36, 37]. Although EE did not completely reverse the decline of newborn neurons after LPS exposure, we found that the decrease in newborn neurons in LPS-exposed mice was further aggregated by the IE. Taken together, the impaired neurogenesis may explain the reasons why the IE has the most negative detrimental effects on cognitive impairment. Our data supported the notion that the potential benefits of EE and alerted the probably side effects of adverse environmental factors on cognitive function for LPS-exposed mice.

Taken together, our findings allow speculation that subjects exposed to the same sepsis protocol may be affected to a different extent depending on their subsequent environmental conditions, which highlights the notion that lifestyle, directly having an impact on the brain microenvironment, in rescuing or aggregating cognitive impairment in neuropsychiatric disorder, including SAE. Notably, our study provides additional evidence for the mechanisms underlying different environmental conditions on cognitive impairment in an animal model of SAE. However, future studies are warranted to investigate the impact of the above-mentioned environmental factors on the long-term cognitive impairment by using a clinically relevant or a more severe septic stimulus model such as CLP.

## MATERIALS AND METHODS

### Animals and study protocol

The present study was approved by the Ethics Committee of Jinling Clinical Medical College of Nanjing Medical University, China, and was conducted in accordance with the Guide for the Care and Use of Laboratory Animals from the National Institutes of Health (Bethesda, MD, USA). Two hundred C57BL/6 male mice (3-4 months) were purchased from the Animal Center of Jinling Hospital, Nanjing, China, and were housed in conditions with a 12-h light–dark cycle (lights on at 07:00) at 24 ± 1 °C with free access to water and chow. The diagram of the present study protocol was shown in Figure 1A.

### SAE animal model

We developed a mouse model of SAE by LPS administration, which have been well described in previous studies [4, 5]. Animals were injected intraperitoneally (i.p.) with either LPS (5 mg/kg) (Escherichia coli endotoxin 0111: B4, Sigma, Lot # 064M4125V) or equal volume (0.9%) normal saline (NS). Our preliminary data showed that this dose and route of administration resulted in a hippocampal-dependent cognitive impairment.

### Administration of bromodeoxyuridine (BrdU)

To label proliferating cells, the BrdU (Sigma Chemical Co., St. Louis, MO) was administered (200 mg/kg per day) i.p. in 0.1 M phosphate buffered-saline (PBS) at pH 7.3 for 5 consecutive days, beginning one week after the start of different environmental condition placement. Animals were sacrificed 3 weeks after the first BrdU injection. This time point was selected because by this time most BrdU-labeled cells represent markers of mature neurons [38, 39].

### Housing environmental conditions

Mice were randomly assigned into three different environmental conditions: SE, EE, or IE, and kept in these housing conditions for four weeks. Standard environment (SE) consisted of common cage housing (25 cm × 20 cm × 15 cm). The EE consisted of larger (60 cm × 35 cm × 20 cm) cages containing constantly a small house and a running wheel for voluntary exercise, and four to five toys that were changed three times a week with new toys of different shape and color. Mice in the SE and EE groups were housed in groups of 4-6, while mice in the IE group were housed singly in individual cages (25 cm × 20 cm × 15 cm) with physical and psychological stresses, including physical restraints for 2 h daily, sleep deprivation due to disturbances biological rhythm induced by noise for 1 h daily (white noise, 3000 HZ, 100 db) and light on overnight.

### Neurobehavioral tests

Behavioral tests were performed successively by the open field, novel object recognition, and fear conditioning tests (n =10-14). All apparatus used in tests were purchased from the Shanghai Softmaze Information Technology Co., Ltd., China. The behavior of mice was recorded using a video camera. A well trained investigator who was blinded to the animal grouping performed the behavioral tests. At the end of each test, the arena was cleaned with 75% alcohol to avoid the presence of olfactory cues. In the present study, each animal underwent three different behavioral tests.

Open field test was performed at 22 day after LPS or NS administration to evaluate the exploratory behavior and anxiety behavior. Mice were placed individually in the center of the black plastic chamber (50 cm × 50 cm × 40 cm) and left free to explore it for 5 min. The total distance traveled and time spent in the center of the open arena was scored by an observer blind to the animal grouping.

Novel object recognition test was conducted at day 24 after LPS or NS administration to evaluate retention or intact memory and exploratory behavior as previously detailed [40]. This test consisted of two trials. In the first (training) trial, two familiar objects were presented. The second trial (testing) with one familiar object and one novel object present in the respective zones of the open field, with 60-min intervals between trials, during which the animals were placed back to their home cages. The time spent with each object was recorded, and the cognitive outcomes were determined by the “discrimination index” for the second trial, which was calculated using the following formula: % discrimination index = Time spent in novel object zone × 100/(time spent in familiar object zone + time spent in novel object zone).

Fear conditioning tests were assessed at days 27-28 after LPS or NS administration to evaluate both hippocampcal and non-hippocampal-dependent cognitive impairment as we described previously [6]. Mice were placed into the conditioning chamber (32 cm × 25 cm × 25 cm), with a stainless steel shock grid floor. The mice were allowed to explore for 3 min for habituation, then a 30 s, 80 dB, 1 kHz tone (CS), which co-terminated with a 2 s, 1.0 mA foot shock (US) was delivered through stainless steel bars by a constant current generator. Two CS-US pairings were separated by a 30-second pause. The contextual memory was tested 24 h after the training. The animals were placed back to the original training chamber to monitor freezing behavior, which was defined as an absence of any movements for more than 3 seconds) was measured. The cued fear memory was tested 2 h later in a novel context with a continuous 3 min training tone presentation to monitor freezing behavior.

### Enzyme-linked immunosorbent assay (ELISA)

The concentrations of tumor necrosis factor (TNF-α), interleukin-1β (IL-1β), IL-6, IL-10, and brain derived neurotrophic factor (BDNF) were determined at 28 day after LPS or NS administration. Mice were killed by an i.p. injection of 2% sodium pentobarbitone (60 mg/kg) and then the prefrontal cortex and hippocampus were collected, then separated, and placed in a homogenizer. The tissues were homogenized with 1 ml ice-cold physiological saline per 100 mg brain tissue. Hypothermal centrifugation was performed at 10,000 × g for 10 min and the supernatant was obtained. The quantifications of TNF-α, IL-1β, IL-6, IL-10, and BDNF were done following the instructions of the manufacture (North China Institute of Biotechnology, Beijing, China).

### Western blotting

The normalized protein samples were subjected to sodium dodecyl sulfate polyacrylamide gel electrophoresis and then were transferred onto polyvinylidene difluoride membranes. Membranes were blocked with 5% skim milk in Tris-buffered saline tween for 1 h and then incubated with anti-IBA1 (1:1000; Abcam, Cambridge, UK), anti-GFAP (1:500; Santa Cruz Biotechnology, Dallas, USA), anti-BDNF (1:1000; Cell Signaling Technology, Boston, USA), and anti-GADPH (1:1000; Cell Signaling Technology, Boston, USA) overnight at 4 °C temperature room. After thorough washing, membranes were incubated in Tris-buffered saline tween with the secondary antibody diluted 1:1000 for 1 h at room temperature. The bands were detected with Pierce ECL Western Blotting Substrate (Thermo Fisher Scientific, Rockford, IL, USA) and semiquantified with image J software (National Institutes of Health, Bethesda, MD, USA).

### BrdU staining

For BrdU immunofluorescence, sections were denatured in 2 M HCl in TBS for 30 min, rinsed and incubated with mouse anti-BrdU (1:100, OBT-0030, Oxford Biotechnology, UK) for 2 d. Then sections were rinsed, incubated with biotinylated anti-rat (1:250; Vector) for 90 min, rinsed, and incubated for 30 min in the dark with streptavidin-conjugated Alexa 568 (1:1,000; Molecular Probes) to visualize BrdU. The analysis was performed using confocal images from coronal sections at similar rostro-caudal levels obtained with a Leica TCS SP5 laser confocal microscopy. An experimenter blinded to the treatment groups counted the BrdU+ cells in the subgranular zones and granule cell layers. The positive cells were counted on sets of every sixth section (six sections per rat) through the rostral-caudal extent of the hippocampal DG.

### Golgi staining

Sample preparation and Golgi silver staining were performed with the FD Rapid Golgi Stain Kit (FD Neurotechnologies, Columbia, MD, USA). Dendritic spine (spine number per 10 μm) for each neuron was analyzed using MATLAB software (MathWorks, Nedik, MA, USA). The spines were counted on two or three segments of secondary dendrites.

### Statistical analysis

Data analysis was performed using SPSS for Windows software (Version 16.0; SPSS, Chicago, IL). Data were presented as mean ± standard error of measurement (S.E.M). Normal distribution of data was analyzed using the Kolmogorov-Smirnov test. Multiple comparisons were analyzed by one-way analysis of variance (ANOVA) followed by post hoc Tukey test. Group comparisons with regard to weight were tested by two-way repeated measures ANOVA followed by Bonferroni test. Since the data of the novel object recognition test are not normally distributed, the Kruskal-Wallis H and Mann-Whitney U tests were used. The survival rate was estimated by Kaplan–Meier method and compared by the log-rank test. A *P* < 0.05 was considered statistically significant.

## References

[1] Iwashyna TJ, Ely EW, Smith DM, Langa KM (2010). Long-term cognitive impairment and functional disability among survivors of severe sepsis. JAMA.

[2] Girard TD, Jackson JC, Pandharipande PP, Pun BT, Thompson JL, Shintani AK, Gordon SM, Canonico AE, Dittus RS, Bernard GR, Ely EW (2010). Delirium as a predictor of long-term cognitive impairment in survivors of critical illness. Crit Care Med.

[3] Pandharipande PP, Girard TD, Jackson JC, Morandi A, Thompson JL, Pun BT, Brummel NE, Hughes CG, Vasilevskis EE, Shintani AK, Moons KG, Geevarghese SK, Canonico A (2013). BRAIN-ICU Study Investigators. Long-term cognitive impairment after critical illness. N Engl J Med.

[4] Anderson ST, Commins S, Moynagh PN, Coogan AN (2015). Lipopolysaccharide-induced sepsis induces long-lasting affective changes in the mouse. Brain Behav Immun.

[5] Czerniawski J, Guzowski JF (2014). Acute neuroinflammation impairs context discrimination memory and disrupts pattern separation processes in hippocampus. J Neurosci.

[6] Wu J, Dong L, Zhang M, Jia M, Zhang G, Qiu L, Ji M, Yang J (2013). Class I histone deacetylase inhibitor valproic acid reverses cognitive deficits in a mouse model of septic encephalopathy. Neurochem Res.

[7] Winocur G (1998). Environmental influences on cognitive decline in aged rats. Neurobiol Aging.

[8] Fuchs F, Cosquer B, Penazzi L, Mathis C, Kelche C, Majchrzak M, Barbelivien A (2016). Exposure to an enriched environment up to middle age allows preservation of spatial memory capabilities in old age. Behav Brain Res.

[9] Lehmann ML, Herkenham M (2011). Environmental enrichment confers stress resiliency to social defeat through an infralimbic cortex-dependent neuroanatomical pathway. J Neurosci.

[10] Chauvet C, Lardeux V, Goldberg SR, Jaber M, Solinas M (2009). Environmental enrichment reduces cocaine seeking and reinstatement induced by cues and stress but not by cocaine. Neuropsychopharmacology.

[11] Volkers KM, Scherder EJ (2011). Impoverished environment, cognition, aging and dementia. Rev Neurosci.

[12] Pickles AR, Hagan JJ, Jones DN, Hendrie CA (2012). Short-term individual housing induced social deficits in female Mongolian gerbils: attenuation by chronic but not acute imipramine. Neuropharmacology.

[13] Jackson JC, Pandharipande PP, Girard TD, Brummel NE, Thompson JL, Hughes CG, Pun BT, Vasilevskis EE, Morandi A, Shintani AK, Hopkins RO, Bernard GR, Dittus RS, Ely EW (2014). Bringing to light the Risk Factors And Incidence of Neuropsychological dysfunction in ICU survivors (BRAIN-ICU) study investigators. Depression, post-traumatic stress disorder, and functional disability in survivors of critical illness in the BRAIN-ICU study: a longitudinal cohort study. Lancet Respir Med.

[14] Wintermann GB, Brunkhorst FM, Petrowski K, Strauss B, Oehmichen F, Pohl M, Rosendahl J (2015). Stress disorders following prolonged critical illness in survivors of severe sepsis. Crit Care Med.

[15] Caruso P, Guardian L, Tiengo T, Dos Santos LS, Junior PM (2014). ICU architectural design affects the delirium prevalence: a comparison between single-bed and multibed rooms. Crit Care Med.

[16] Montarolo F, Parolisi R, Hoxha E, Boda E, Tempia F (2013). Early enriched environment exposure protects spatial memory and accelerates amyloid plaque formation in APP(Swe)/PS1(L166P) mice. PLoS One.

[17] Areán PA, Raue P, Mackin RS, Kanellopoulos D, McCulloch C, Alexopoulos GS (2010). Problem-solving therapy and supportive therapy in older adults with major depression and executive dysfunction. Am J Psychiatry.

[18] Wang XD, Rammes G, Kraev I, Wolf M, Liebl C, Scharf SH, Rice CJ, Wurst W, Holsboer F, Deussing JM, Baram TZ, Stewart MG, Müller MB, Schmidt MV (2011). Forebrain CRF_1_ modulates early-life stress-programmed cognitive deficits. J Neurosci.

[19] de Macedo LD, De Oliveira TC, Soares FC, Bento-Torres J, Bento-Torres NV, Anthony DC, Picanço-Diniz CW (2015). Beneficial effects of multisensory and cognitive stimulation in institutionalized elderly: 12-months follow-up. Clin Interv Aging.

[20] Possamai F, dos Santos J, Walber T, Marcon JC, dos Santos TS, Lino de, Oliveira C (2015). Influence of enrichment on behavioral and neurogenic effects of antidepressants in Wistar rats submitted to repeated forced swim test. Prog Neuropsychopharmacol Biol Psychiatry.

[21] Green D, Charman T, Pickles A, Chandler S, Loucas T, Simonoff E, Baird G (2009). Impairment in movement skills of children with autistic spectrum disorders. Dev Med Child Neurol.

[22] Czerniawski J, Miyashita T, Lewandowski G, Guzowski JF (2015). Systemic lipopolysaccharide administration impairs retrieval of context-object discrimination, but not spatial, memory: evidence for selective disruption of specific hippocampus-dependent memory functions during acute neuroinflammation. Brain Behav Immun.

[23] Freret T, Billard JM, Schumann-Bard P, Dutar P, Dauphin F, Boulouard M, Bouet V (2012). Rescue of cognitive aging by long-lasting environmental enrichment exposure initiated before median lifespan. Neurobiol Aging.

[24] Leger M, Paizanis E, Dzahini K, Quiedeville A, Bouet V, Cassel JC, Freret T, Schumann-Bard P, Boulouard M (2015). Environmental enrichment duration differentially affects behavior and neuroplasticity in adult mice. Cereb Cortex.

[25] McKim DB, Niraula A, Tarr AJ, Wohleb ES, Sheridan JF, Godbout JP (2016). Neuroinflammatory dynamics underlie memory impairments after repeated social defeat. J Neurosci.

[26] Osso LA, Chan JR (2015). Astrocytes underlie neuroinflammatory memory impairment. Cell.

[27] Ji MH, Qiu LL, Tang H, Ju LS, Sun XR, Zhang H, Jia M, Zuo ZY, Shen JC, Yang JJ (2015). Sepsis-induced selective parvalbumin interneuron phenotype loss and cognitive impairments may be mediated by NADPH oxidase 2 activation in mice. J Neuroinflammation.

[28] Verma R, Friedler BD, Harris NM, McCullough LD (2014). Pair housing reverses post-stroke depressive behavior in mice. Behav Brain Res.

[29] Jurgens HA, Johnson RW (2012). Environmental enrichment attenuates hippocampal neuroinflammation and improves cognitive function during influenza infection. Brain Behav Immun.

[30] Williamson LL, Chao A, Bilbo SD (2012). Environmental enrichment alters glial antigen expression and neuroimmune function in the adult rat hippocampus. Brain Behav Immun.

[31] Snigdha S, Prieto GA, Petrosyan A, Loertscher BM, Dieskau AP, Overman LE, Cotman CW (2016). H3K9me3 inhibition improves memory, promotes spine formation, and increases BDNF levels in the aged hippocampus. J Neurosci.

[32] Lieberwirth C, Pan Y, Liu Y, Zhang Z, Wang Z (2016). Hippocampal adult neurogenesis: its regulation and potential role in spatial learning and memory. Brain Res.

[33] Hullinger R, O'Riordan K, Burger C (2015). Environmental enrichment improves learning and memory and long-term potentiation in young adult rats through a mechanism requiring mGluR5 signaling and sustained activation of p70s6k. Neurobiol Learn Mem.

[34] Novkovic T, Mittmann T, Manahan-Vaughan D (2015). BDNF contributes to the facilitation of hippocampal synaptic plasticity and learning enabled by environmental enrichment. Hippocampus.

[35] Schloesser RJ, Lehmann M, Martinowich K, Manji HK, Herkenham M (2010). Environmental enrichment requires adult neurogenesis to facilitate the recovery from psychosocial stress. Mol Psychiatry.

[36] Besnard A, Sahay A (2016). Adult hippocampal neurogenesis, fear generalization, and stress. Neuropsychopharmacology.

[37] Parihar VK, Hattiangady B, Shuai B, Shetty AK (2013). Mood and memory deficits in a model of Gulf War illness are linked with reduced neurogenesis, partial neuron loss, and mild inflammation in the hippocampus. Neuropsychopharmacology.

[38] Mirescu C, Peters JD, Gould E (2004). Early life experience alters response of adult neurogenesis to stress. Nat Neurosci.

[39] Leal-Galicia P, Saldívar-González A, Morimoto S, Arias C (2007). Exposure to environmental enrichment elicits differential hippocampal cell proliferation: role of individual responsiveness to anxiety. Dev Neurobiol.

[40] Zhang MQ, Ji MH, Zhao QS, Jia M, Qiu LL, Yang JJ, Peng YG, Yang JJ, Martynyuk AE (2015). Neurobehavioural abnormalities induced by repeated exposure of neonatal rats to sevoflurane can be aggravated by social isolation and enrichment deprivation initiated after exposure to the anaesthetic. Br J Anaesth.

